# Evaluation of α‐synuclein in CNS‐originating extracellular vesicles for Parkinsonian disorders: A systematic review and meta‐analysis

**DOI:** 10.1111/cns.14341

**Published:** 2023-07-07

**Authors:** Hash Brown Taha, Shomik S. Ati

**Affiliations:** ^1^ Department of Integrative Biology & Physiology University of California Los Angeles Los Angeles California USA

**Keywords:** Diagnosis, extracellular vesicles, L1CAM, Parkinson's disease, α‐Synuclein

## Abstract

**Background & Aims:**

Parkinsonian disorders, such as Parkinson's disease (PD), multiple system atrophy (MSA), dementia with Lewy bodies (DLB), progressive supranuclear palsy (PSP) and corticobasal syndrome (CBS), share early motor symptoms but have distinct pathophysiology. As a result, accurate premortem diagnosis is challenging for neurologists, hindering efforts for disease‐modifying therapeutic discovery. Extracellular vesicles (EVs) contain cell‐state‐specific biomolecules and can cross the blood‐brain barrier to the peripheral circulation, providing a unique central nervous system (CNS) insight. This meta‐analysis evaluated blood‐isolated neuronal and oligodendroglial EVs (nEVs and oEVs) α‐synuclein levels in Parkinsonian disorders.

**Methods:**

Following PRISMA guidelines, the meta‐analysis included 13 studies. An inverse‐variance random‐effects model quantified effect size (SMD), QUADAS‐2 assessed risk of bias and publication bias was evaluated. Demographic and clinical variables were collected for meta‐regression.

**Results:**

The meta‐analysis included 1,565 patients with PD, 206 with MSA, 21 with DLB, 172 with PSP, 152 with CBS and 967 healthy controls (HCs). Findings suggest that combined concentrations of nEVs and oEVs α‐syn is higher in patients with PD compared to HCs (SMD = 0.21, p = 0.021), while nEVs α‐syn is lower in patients with PSP and CBS compared to patients with PD (SMD = ‐1.04, p = 0.0017) or HCs (SMD = ‐0.41, p < 0.001). Additionally, α‐syn in nEVs and/or oEVs did not significantly differ in patients with PD vs. MSA, contradicting the literature. Meta‐regressions show that demographic and clinical factors were not significant predictors of nEVs or oEVs α‐syn concentrations.

**Conclusion:**

The results highlight the need for standardized procedures and independent validations in biomarker studies and the development of improved biomarkers for distinguishing Parkinsonian disorders.

## Graphical abstract

Parkinsonian disorders share Parkinsonism symptoms but differ pathophysiologically. This meta‐analysis investigates α‐synuclein concentrations in CNS‐originating EVs, revealing only higher α‐syn in Parkinson's disease compared to healthy controls and progressive supranuclear palsy and corticobasal syndrome, but not multiple system atrophy and dementia with Lewy body, emphasizing the necessity for improved biomarkers and standardized methodologies.

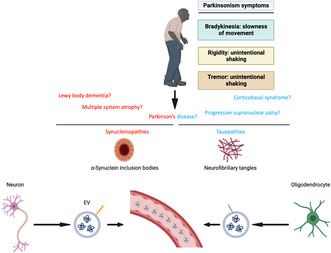

## 1 INTRODUCTION

Parkinsonian disorders encompass a group of neurodegenerative conditions characterized by motor symptoms such as bradykinesia, rigidity, and tremor, among which Parkinson's disease (PD) is the most prevalent.^1^ Other less common but clinically significant Parkinsonian disorders include multiple system atrophy (MSA), dementia with Lewy body (DLB), progressive supranuclear palsy (PSP), and corticobasal degeneration (CBD). Despite the common prevalence of shared symptoms, these disorders differ in the types of cells affected and the specific regions of the brain involved and do not respond to the same treatments.^2^

PD, MSA and DLB share a common pathological hallmark in the form of abnormal α‐synuclein (α‐syn) aggregation. In PD, α‐syn aggregates primarily form in dopaminergic neurons of the substantia nigra, causing their degeneration and the resulting motor symptoms.^3^, ^4^ In contrast, DLB presents with α‐syn inclusions predominantly in cortical neurons, leading to cognitive and psychiatric manifestations.^5^ MSA, on the other hand, features α‐syn accumulation in both neurons and oligodendrocytes, known as glial cytoplasmic inclusions (GCIs), which affect various brain regions, including the basal ganglia, cerebellum, and brainstem.^6^

Additionally, PSP and CBD share the presence of tau protein pathology, but their regional distribution differs. PSP is characterized by the accumulation of hyperphosphorylated tau proteins in both neurons and glial cells, primarily affecting the brainstem, basal ganglia, and cerebellum, leading to a range of motor, cognitive, and oculomotor deficits.^7^ In CBD, the abnormal deposition of tau proteins is more localized, predominantly affecting the frontoparietal cortex and basal ganglia asymmetrically, causing varied motor, cognitive and sensory symptoms.^8^ In this review, we utilize the term corticobasal syndrome (CBS) instead of CBD based on clinical assessments or diagnostic criteria employed in the included studies, as definitive postmortem confirmation of the diagnosis was not available.

These differences in cellular and regional involvement of α‐syn and tau proteoforms, different molecular variants of the protein such as post‐translationally modified oligomers, underscore the distinct pathophysiological mechanisms at play in PD, DLB, MSA, PSP, and CBD/CBS. Despite these differences, they are frequently misdiagnosed due to overlapping clinical features.^9^ Recognizing these nuances is crucial for accurate diagnosis and tailored therapeutic approaches in managing these complex neurodegenerative conditions.

Extracellular vesicles (EVs) are small membrane‐bound structures released by cells that play critical roles in intercellular communication and the regulation of various physiological processes. These vesicles encapsulate a wide range of biomolecules, including proteins, lipids, and nucleic acids, which reflect the state of the parent cell.^10^ Due to their ability to cross the blood–brain barrier, EVs offer a unique window into the brain's biochemistry, allowing for the study of central nervous system (CNS) processes and potential biomarker discovery in neurological disorders.^11^, ^12^, ^13^ By carrying cell‐state‐specific messages from the CNS to the peripheral circulation, EVs have emerged as an avenue for minimally invasive diagnostic and therapeutic approaches in neurodegenerative diseases^14^ and Parkinsonian disorders.^12^, ^15^, ^16^, ^17^, ^18^, ^19^, ^20^, ^21^, ^22^, ^23^, ^24^, ^25^, ^26^

Since 2014, many studies have employed the approach of measuring α‐syn in neuronal and oligodendroglial extracellular vesicles (nEVs and oEVs) to differentially diagnose Parkinsonian disorders from one another or from healthy controls (HCs). Two recent meta‐analyses compared α‐syn levels in putative “exosomes” in patients with PD,^27^, ^28^ but they had different objectives than the current meta‐analysis. These meta‐analyses grouped α‐syn concentrations from EVs isolated from various sources, which are known to be incomparable, omitted at least six studies with CNS‐originating EV α‐syn measurements in PD, and did not conduct separate analyses for patients with PD against other Parkinsonian disorders or patients with other Parkinsonian disorders against HCs. Additionally, they did not take into consideration key aspects in the literature known to affect CNS‐originating EVs' downstream protein analyses.^29^, ^30^

Therefore, this systematic review and meta‐analysis aimed to compile the evidence regarding α‐syn proteoform levels in isolated nEVs and oEVs from patients with Parkinsonian disorders or HCs. Furthermore, we assessed whether demographic or clinical variables influenced nEVs and/or oEVs α‐syn using meta‐regressions.

## 2 METHODOLOGY

We conducted a systematic review, meta‐analysis and meta‐regression according to the protocol recommended by the Preferred Reporting Items for Systematic Reviews and Meta‐Analyses Protocols (PRISMA). Our study only involved anonymized data, and we did not collect any personal information or perform any procedures on human subjects. Therefore, ethical approval was not necessary. We did not register the study's protocol.

### 2.1 Data sources and search strategy

We performed a thorough search for relevant articles by using specific search terms related to PD and Parkinsonian disorders. The search was conducted in two databases (PUBMED and EMBASE) and covered articles published from the inception of the databases until March 26, 2023. The search terms we used included combinations of “Parkinson's disease,” “Lewy body dementia,” “dementia with Lewy body,” “Parkinson's disease dementia,” “multiple system atrophy,” “progressive supranuclear palsy,” “corticobasal syndrome,” “corticobasal degeneration,” “parkinsonism,” “synuclein,” “neuronal EVs,” “neuronal exosomes,” “extracellular vesicles,” “exosomes,” “blood,” “serum,” “plasma,” and “cerebrospinal fluid.”. Two independent researchers (HBT and SSA) screened all the titles, abstracts, and full manuscripts to select articles that met the eligibility criteria. By hand, we reviewed the reference lists of eligible studies, searched Google scholar for articles measuring α‐syn in CNS‐originating EVs and detailed reviews of the literature for inclusion of eligible studies.^29^, ^30^, ^31^, ^32^, ^33^, ^34^, ^35^ Any discrepancies in article selection were resolved through discussion. The complete search strategy can be found in Table S1.

### 2.2 Eligibility criteria

The eligible studies had to assess the levels of α‐syn in CNS‐originating EVs obtained from plasma, serum, or the cerebrospinal fluid in patients with PD along with at least one of the following diseases: MSA, DLB, PSP, CBS, or HCs. We excluded studies that used animals or cell lines, studies that did not include the specified diseases and studies that did not report the sample size. If α‐syn levels were not included in the study, we reached out to the authors to obtain the mean ± standard deviation (SD). For studies that included longitudinal measurements or treatment interventions, we only considered the baseline assessments. For studies that included more than one cohort, all cohorts were averaged. For studies that included subgroup analyses for the diseases (e.g., PD + dementia, PD + mild‐cognitive impairment, etc.,), all values were averaged and included together.

### 2.3 Data extraction

Data from eligible studies were extracted by two independent researchers (HBT and SSA) using a standardized form. Both authors checked the database for accuracy and completeness, and the extracted data included the following information: first author's surname, publication year, medium (plasma or serum), EV isolation methodology, antibody marker and clone for enrichment nEVs or oEVs, EV characterization and quantification methodology, analytical method, mean ± SD of α‐syn proteoforms, number of patients in each group, age, female percentage, disease duration, disease stage (Hoehn & Yahr scale), motor severity (UPDRSIII or UMSARS), and cognitive score (MMSE or MoCA). If any information was missing, we contacted the corresponding author to obtain the information. In one study,^21^ the values were given as median (IQR), and the mean and SD were estimated using established statistical formulas,^36^, ^37^ “Mean ≈ Median + 1.35 * (IQR/2)” and “SD ≈ IQR/1.35”.

### 2.4 Risk of bias assessment

We assessed the quality and risk of bias of all eligible studies using the Quality Assessment for Diagnostic Accuracy Studies (QUADAS‐2) criteria.^38^ Independent researchers (HBT and SSA) conducted the quality assessment and any discrepancies were resolved through discussion until a consensus was reached. More information about the quality assessment is available in Table S2.

### 2.5 Data synthesis and statistics

Meta‐analyses were performed in R software (version 2022.12.0+353). We examined the differences of nEVs and oEVs α‐syn among patients with PD, MSA, DLB, PSP and CBS versus HCs together and separately. Pooled standardized mean differences and 95% CIs were calculated based on Cohen's *d* ^36^ using the below formula:

$$\text{Pooled standard deviation}\;\left(\mathrm{SD}\right)=\sqrt{\left(\frac{\left(\left(n1-1\right)*\mathrm{SD}1^{2}+\left(n2-1\right)*\mathrm{SD}2^{2}\right)}{n1+n2-2}\right)}$$

$$\text{Standardized mean difference}\;\left(\mathrm{SMD}\right)=\frac{\left(M1-M2\right)}{\text{Pooled}\;\mathrm{SD}}$$

Funnel plots, Begg's rank correlation and Egger's regression tests were used to evaluate publication bias.^39^ Meta‐analyses were based on a random effects model with inverse‐sample weighting. In cases with high heterogeneity, sensitivity analyses were conducted to repeat the analysis with the exclusion of highly influential studies. Meta‐regressions were used to investigate whether age, gender, disease duration, motor impairment and cognition severity were acting as covariates. We excluded disease stage from the meta‐regression due to a significantly high correlation with motor impairment severity (*r* = 0.66, *p* < 0.001).

## 3 RESULTS

**FIGURE 1:**
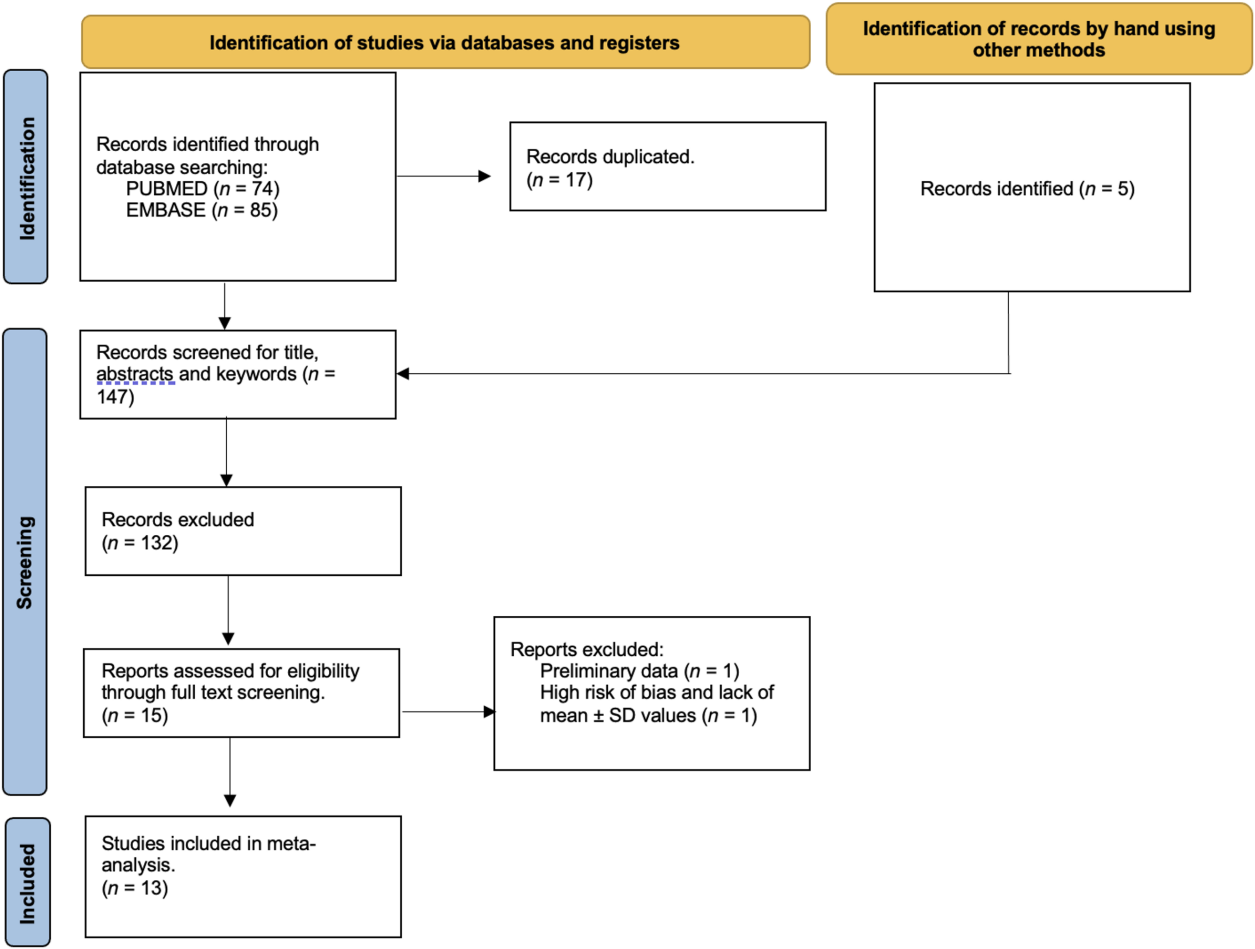
PRISMA flow diagram for study inclusion or exclusion strategy.

The systematic and hand search identified 164 articles of which 17 duplicated articles were removed. After title and abstract screening of 147 articles, 15 articles were considered potentially eligible (Figure 1). One article was removed^40^ because it included only preliminary data. Three articles did not include nEVs or oEVs α‐syn mean ± SD,^12^, ^20^, ^25^ while the mean ± SD in one article was estimated using established statistical formulas (see above).^21^ All authors were contacted to obtain the missing information. One study with high bias quantified nEVs α‐syn using dot blots without a reference to a standard calibrator and did not estimate the α‐syn mean ± SD concentrations.^20^ As such, it was removed from the meta‐analysis. In total, thirteen articles^12^, ^15^, ^16^, ^17^, ^18^, ^19^, ^21^, ^22^, ^23^, ^24^, ^25^, ^26^, ^41^ were included in the meta‐analysis. A detailed description of each individual study is provided in Table 1.

**TABLE 1:** Summary of demographic characteristics of patients with a Parkinsonian disorder or healthy controls (HC) in studies included in the meta‐analysis and meta‐regression.

Study	EV isolation method	CNS‐EV antibody	EV confirmation method	Quantification method	Proteoform	# PD	# MSA	# DLB	# PSP	# CBS	# HC	Age (years)	Female (%)	Disease duration (years)	HY scale	UPDRS III	MMSE	MoCA
*Plasma*																		
Shi et al. 2014^12^	2000× *g* for 15 min followed by 12,000× *g* for 30 min^42^ followed by direct IP	L1CAM (clone UJ127)	EM WB	Luminex (in‐house^43^)	α‐syn	267	0	0	0	0	215	PD: 66.3 ± 9.1 HC: 65.7 ± 9.1	PD: 44.6% HC: 46.0%	9.6 ± 6.6	2.4 ± 0.7	28.4 ± 12.6	28.0 ± 2.6	NA
Zhao et al. 2019^26^	ExoQuick (Systems Biosciences)	L1CAM (clone 5G3)	TEM	Sandwich ELISA (R&D Systems)	α‐syn	39	0	0	0	0	40	PD: 67.5 ± 6.9 HC: 66.6 ± 8.8	PD: 41.0% HC: 57.5%	5.0 ± 3.2	NA	48.6 ± 21.0	NA	NA
Niu et al. 2020^22^	2000× *g* for 15 min followed by 12,000× *g* for 30 min^42^ followed by direct IP	L1CAM (clone UJ127)	TEM TRPS WB	ECLIA (Meso Scale Discovery)	α‐syn	53	0	0	0	0	21	PD: 65.0 ± 5.3 HC: 64.0 ± 5.4	PD: 53.0% HC: 48.0%	NA	2.0 ± 0.5	22.3 ± 10.3	27.6 ± 2.6	23.6 ± 3.6
Zou et al. 2020^41^	2000× *g* for 15 min followed by 12,000× g for 30 min^42^ followed by direct IP	L1CAM (clone UJ127)	TEM NTA WB	Simoa (Quanterix)	α‐syn	93 Early: 51 Advanced: 42	0	0	0	0	85	PD: 66.9 ± 9.5 HC: 66.2 ± 10.3	PD: 43.0% HC: 43.5%	4.2 ± 2.5	2.8 ± 0.5	28.7 ± 16.0	24.3 ± 2.9	NA
Yu et al. 2020^25^	2000× *g* for 15 min followed by 12,000× *g* for 30 min^42^ followed by direct IP	L1CAM (clone UJ127) CNPase (clone mABcam 44289)	NTA TEM WB	Luminex (in‐house^43^)	α‐syn	34	32	0	0	0	31	PD: 63.6 ± 8.0 MSA: 63.0 ± 6.9 HC: 64.3 ± 7.5	PD: 41.2% MSA: 40.6% HC: 51.6%	PD: 4.0 ± 2.2 MSA: 4.0 ± 2.8	NA	PD: 21.4 ± 10.6 MSA (UMSARS): 24.1 ± 10.6	NA	NA
Blommer et al. 2022^16^	ExoQuick (Systems Biosciences)	L1CAM (clone 5G3)	CFM, Cryo‐TEM NTA ExoView WB	ECLIA (Meso Scale Discovery)	α‐syn	224 NC: 103 MCI: 81 PDD: 40	0	0	0	0	49	PD: 71.8 ± 7.0 HC: 75.8 ± 7.3	PD: 30.3% HC: 42.8%	9.3 ± 5.5	2.5 ± 1.0	39.5 ± 14.1	NA	23.6 ± 4.8
*Serum*																		
Si et al. 2019^23^	ExoQuick (Systems Biosciences)	L1CAM (clone UJ127)	EM WB	Sandwich ELISA (CUSABIO)	Oligomeric α‐syn	38	0	0	0	0	18	PD: 62.4 ± 9.7 HC: 62.7 ± 2.3	PD: 50.0% HC: 55.5%	2.3 ± 1.8	1.7 ± 0.56	18.6 ± 10.2	NA	NA
Jiang et al. 2020^19^	Direct IP	L1CAM (clone UJ127)	NTA SEM WB	ECLIA (Meso Scale Discovery)	α‐syn	275 PDD: 45	14	21	35	45	144	PD: 68.9 ± 7.1 MSA: 68.1 ± 10.8 DLB: 68.5 ± 4.9 PSP: 68.0 ± 7.5 CB:61.1 + 7.2 HC:68.1 ± 10.8	PD: 33.8% MSA: 40.0% DLB: 71.4% PSP: 48.6% CBS: 40.0% HC: 34.7%	PD: 7.5 ± 7.0 MSA: 4.9 ± 2.6 DLB: 3.4 ± 3.0 PSP: 2.8 ± 1.8 CBS: 1.9 ± 1.3	NA	PD: 32.2 ± NA MSA: 27.7 ± NA DLB: 20.9 ± NA PSP: 24.5 ± NA CBS: 22.5 ± NA	NA	PD: 22.7 ± NA MSA: 16.9 ± NA DLB: 16.3 ± NA PSP: 21.4 ± NA CBS: 22.3 ± NA
Agliardi et al. 2021^15^	ExoQuick (Systems Biosciences)	L1CAM (clone 5G3)	Exo‐Check Antibody Array NTA TEM WB	Sandwich ELISA (SNCOα; MyBiosource)	Oligomeric α‐syn	32	0	0	0	0	40	PD: 69.5 ± 8.6 HC: 57.4 ± 7.6	PD: 34.4% HC: 47.5%	6.3 ± 3.6	2.0 ± NA	28.5 ± 13.2	NA	24.2 ± 2.5
Jiang et al. 2021^18^	Direct IP	L1CAM (clone UJ127)	NTA SEM WB	ECLIA (Meso Scale Discovery)	α‐syn	290	50	0	116	88	191	PD: 65.1 ± 7.8 MSA: 67.1 ± 10.0 PSP: 69.5 ± 2.2 CBS: 64.6 ± 7.2 HC: 64.4 ± 6.8	PD: 34.8% MSA: 30.0% PSP: 37.9% CBS: 53.4% HC: 41.9%	PD: 7.4 ± 3.1 MSA: 5.2 ± 2.7 PSP: 3.5 ± 2.2 CBS: 3.3 ± 2.0	NA	PD: 25.9 ± NA MSA: 27.7 ± NA PSP: 31.0 ± NA CBS: 36.1	NA	PD: 26.8 ± NA MSA: 26.0 ± NA PSP: 22.0 ± NA CBS: 20.9 ± NA
Dutta et al. 2021^17^	ExoQuick (Systems Biosciences)	L1CAM (clone 5G3) MOG (clone D‐2)	FC TEM TRPS WB	ECLIA (Meso Scale Discovery)	α‐syn	104	80	0	0	0	101	PD: 66.8 ± 9.3 MSA: 62.8 ± 8.1 HC: 64.9 ± 10.5	PD: 55.5% MSA: 51.2% HC: 55.4%	PD: 7.0 ± 4.4 MSA: 5.0 ± 2.8	PD: 2.4 ± 0.9 MSA: 3.8 ± 1.9	PD: 20.7 ± 14.3 MSA: NA	PD: 27.0 ± 4.2 MSA 27.2 ± 5.5	NA
Meloni et al. 2023^21^	ExoQuick (Systems Biosciences)	L1CAM (clone 5G3)	Exo‐Check Antibody Array NTA TEM WB	Sandwich ELISA (SNCOα; MyBiosource)	Oligomeric α‐syn	70	0	0	21	19	0	PD: 69.5 ± 7.5 PSP: 72.8 ± 8.5 CBS: 71.9 ± 8.0	PD: 44.2% PSP: 47.6% CBS: 57.9%	PD: 7.3 ± 5.6 PSP: 4.0 ± 1.6 CBS: 4.4 ± 3.1	PD: 2.1 ± 0.6 PSP: NA CBS: NA	PD: 33.0 ± 14.4 PSP: NA CBS: NA	NA	PD: 25.1 ± 2.7 PSP: 17.8 ± 5.1 CBS: 17.0 ± 8.1
Taha et al. 2023^24^	ExoQuick (Systems Biosciences)	L1CAM (clone 5G3) MOG (clone D‐2)	FC TEM TRPS WB	ECLIA (in‐house^44^)	pS129‐α‐syn	46	30	0	0	0	32	PD: 66.8 ± 11.6 MSA: 62.7 ± 8.2	PD: 46.8% MSA: 56.7%	PD: 8.1 ± 5.0 MSA: 62.7 ± 8.2	PD: 2.5 ± 1.0 MSA: 3.8 ± 1.0	PD: 25.1 ± 15.6 MSA: NA	PD: 26.3 ± 6.4 MSA: 26.5 ± 9.3	NA

Abbreviations: CBS, corticobasal syndrome; CFM, confocal fluorescence microscopy; CNPase, 2′,3′‐cyclic‐nucleotide 3′‐phosphodiesterase; DLB, dementia with Lewy body; ECLIA, electrochemilumiscence ELISA; ELISA, Enzyme‐linked immunosorbent assay; EM, electron microscopy; EV, extracellular vesicle; FC, flow‐cytometry; HC, healthy control; HY, Hoehn and Yahr disease stage scale^45^; IP, immunoprecipitation; L1CAM, L1 cell adhesion molecule; MCI, mild cognitive impairment; MMSE, Mini‐mental state examination^46^; MoCA, Montreal cognitive assessment^47^; MOG, myelin oligodendrocyte glycoprotein; MSA, multiple system atrophy; NC, non‐cognitively impaired; NTA, nanoparticle tracking analysis; PD, Parkinson's disease; PDD, PD with dementia; pS129‐α‐syn, phosphorylated α‐syn at Ser 129; PSP, progressive supranuclear palsy; SEM, scanning EM; TEM, transmission EM; TRPS, tunable resistive pulse sensing; UMSARS, unified multiple system atrophy rating scale^48^; UPDRSIII, Unified Parkinson's disease rating scale.^49^; WB, Western blot.

### 3.1 Study characteristics

In total, the meta‐analysis included 1,565 patients with PD, 206 with MSA, 21 with DLB, 172 with PSP, 152 with CBS and 967 HCs. All patients included measurements of at least one single proteoform of α‐syn (non‐modified, oligomeric or phosphorylated). Three studies quantified nEVs oligomeric α‐syn in a total of 140 patients with PD and 58 HCs. Two studies quantified oEVs^17^, ^25^ α‐syn in 138 patients with PD, 112 patients with MSA and 132 HCs. One study quantified^24^ nEVs and oEVs phosphorylated α‐syn at Ser 129 (pS129‐α‐syn) in 46 patients with PD, 30 patients with MSA and 32 HCs. The publication year ranged from 2014 to 2023. Most studies quantified proteoforms of α‐syn using electrochemiluminescence (ECLIA) or Sandwich ELISA, while two studies used a Luminex assay^12^, ^25^ and one used a Simoa assay.^41^ Two articles divided the PD population into subgroups of non‐cognitive impairment (*n* = 103), mild cognitive impairment (*n* = 81) and PD with dementia (*n* = 85),^16^, ^19^ while one article^41^ divided PD onto early (*n* = 41) versus late stage (*n* = 52).

The overall quality of the studies included was high (see Table S3). The risk of bias in patient selection for all included studies was unclear, as the sampling method was not reported. No study participant exclusion was determined by any of the studies. As measurement of α‐syn in nEVs and oEVs is an objective measure, the index test domain was deemed to be low bias because prior knowledge of the clinical status (patients with a Parkinsonian disorder or HCs) should not influence the objective measurement of α‐syn. Most of the articles (*n* = 10, 76.9%) had low bias regarding Reference Standard, while three studies^12^, ^24^, ^25^ that have used an in‐house test were deemed as high risk of bias. With respect to Flow and Timing domain, all studies were deemed low risk of bias because the time interval from clinical diagnosis to the index test (i.e., α‐syn proteoforms measurement) could be estimated.

It is important to note that EVs' purity, content, size and number are known to be dependent on a variety of preanalytical factors including the choice of anticoagulation agent mixed with plasma, the time of preparation, centrifugation methodology, the nature of transport, number of freeze/thaw cycles, storage conditions, temperature, and the type of collection tube,^50^, ^51^, ^52^, ^53^, ^54^, ^55^, ^56^ which are not standardized across biobanks or clinical laboratory collection methodologies. Further, the anti‐L1CAM antibody clone UJ127 has come under scrutiny for its ability to cross‐react with α‐syn antibodies.^57^ Therefore, when applicable, for each of the sections below, we conducted subgroup analyses by (1) medium (plasma vs. serum), (2) antibody clone used (i.e., anti‐L1CAM UJ127 vs. 5G3), (3) analytical method, (4) center/cohort and (5) subgroup diagnoses and their variable combinations. To avoid redundancy, subgroup analyses are discussed in detail if they are applicable and show contrasting results to grouped analyses.

As heterogeneity was high in most analyses, we conducted all meta‐analyses using an inverse‐variance random effect model with the restricted maximum likelihood method and performed sensitivity analyses to explore the exclusion of influential studies driving the effect.

### 3.2 nEVs & oEVs α‐syn: Parkinsonian disorders versus healthy controls

**FIGURE 2:**
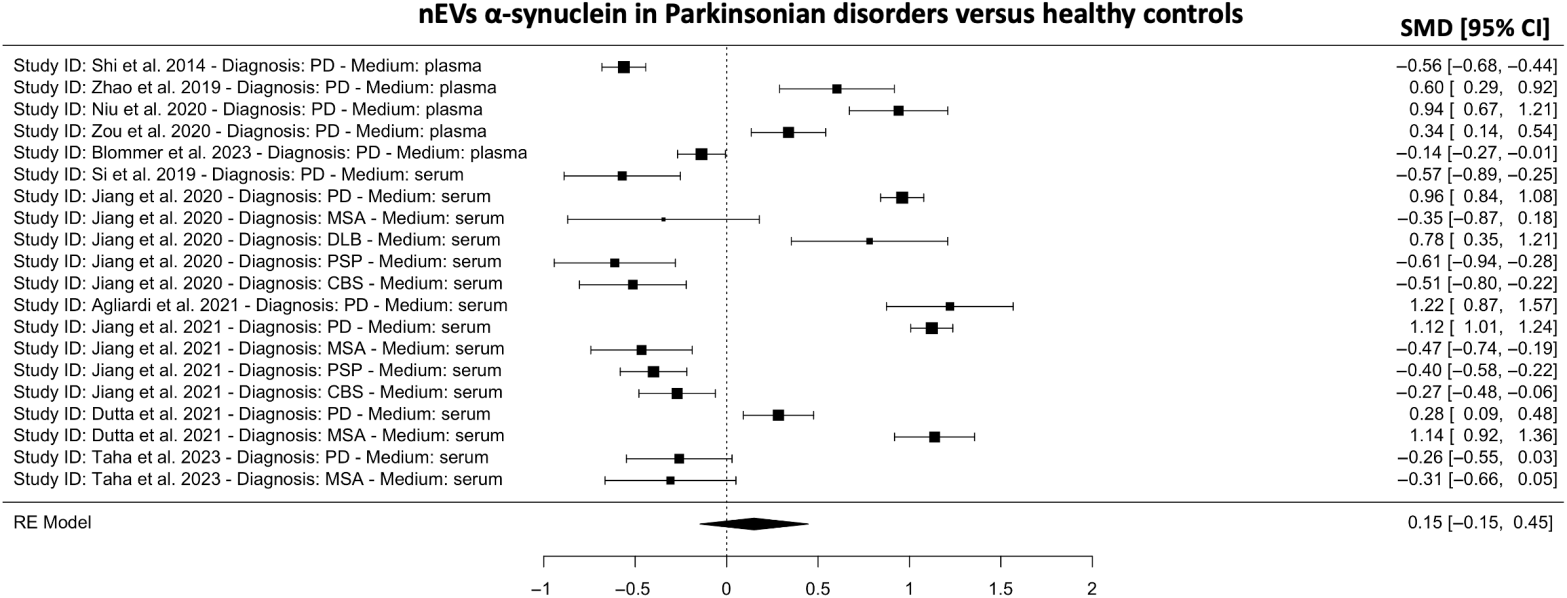
Meta‐analysis for neuronal EVs (nEVs) α‐synuclein in Parkinsonian disorders vs. healthy controls (HC). A positive or negative SMD indicates higher or lower nEVs α‐synuclein concentrations in a Parkinsonian disorder versus HCs, respectively. CBS, corticobasal syndrome; CI, confidence intervals; DLB, dementia with Lewy body; MSA, multiple system atrophy; PD, Parkinson's disease; PSP, progressive supranuclear palsy; SMD, standardized mean difference.

We first asked whether nEVs α‐syn is different in all Parkinsonian disorders versus HCs. The meta‐analysis suggests that nEVs α‐syn is not different in Parkinsonian disorders vs. HCs (*k* = 20, SMD = 0.15; 95% CI −0.15, 0.99; *p* = 0.047; Figure 2). Due to high heterogeneity (97.6%), we conducted sensitivity analyses to identify influential studies. Two studies were identified to be influential.^15^, ^19^ Removal of the studies from the analysis did not alter the results and heterogeneity continued to be high (97.5%). As described above, the medium used for the isolation of EVs and the antibody used for the enrichment of nEVs are known to impact downstream analyses. Separating the analyses by medium and antibody did not alter the results.

Adding α‐syn in oEVs^17^, ^24^, ^25^ to the meta‐analysis did not alter the results, although the p‐value almost reached significance (*k* = 26, SMD = 0.18; 95% CI −0.01, 0.38; *p* = 0.062; Figure S1). This effect remained unchanged after separating the meta‐analysis by medium and antibody.

Moreover, separate analyses for all synucleinopathies (PD, MSA, and DLB) versus HCs revealed non‐significant results (*k* = 16, SMD = 0.30; 95% CI −0.03, 0.63; *p* = 0.073; Figure S2A) even when the analysis was further divided to patients with PD only (*k* = 11, SMD = 0.36; 95% CI −0.04, 0.75; *p* = 0.29; Figure S2B) or MSA only (*k* = 4, SMD = 0.02; 95% CI −0.74, 0.78; *p* = 0.96; Figure S2C). Patients with DLB were included only in one study, and as such, no separate meta‐analysis was conducted.

**FIGURE 3:**
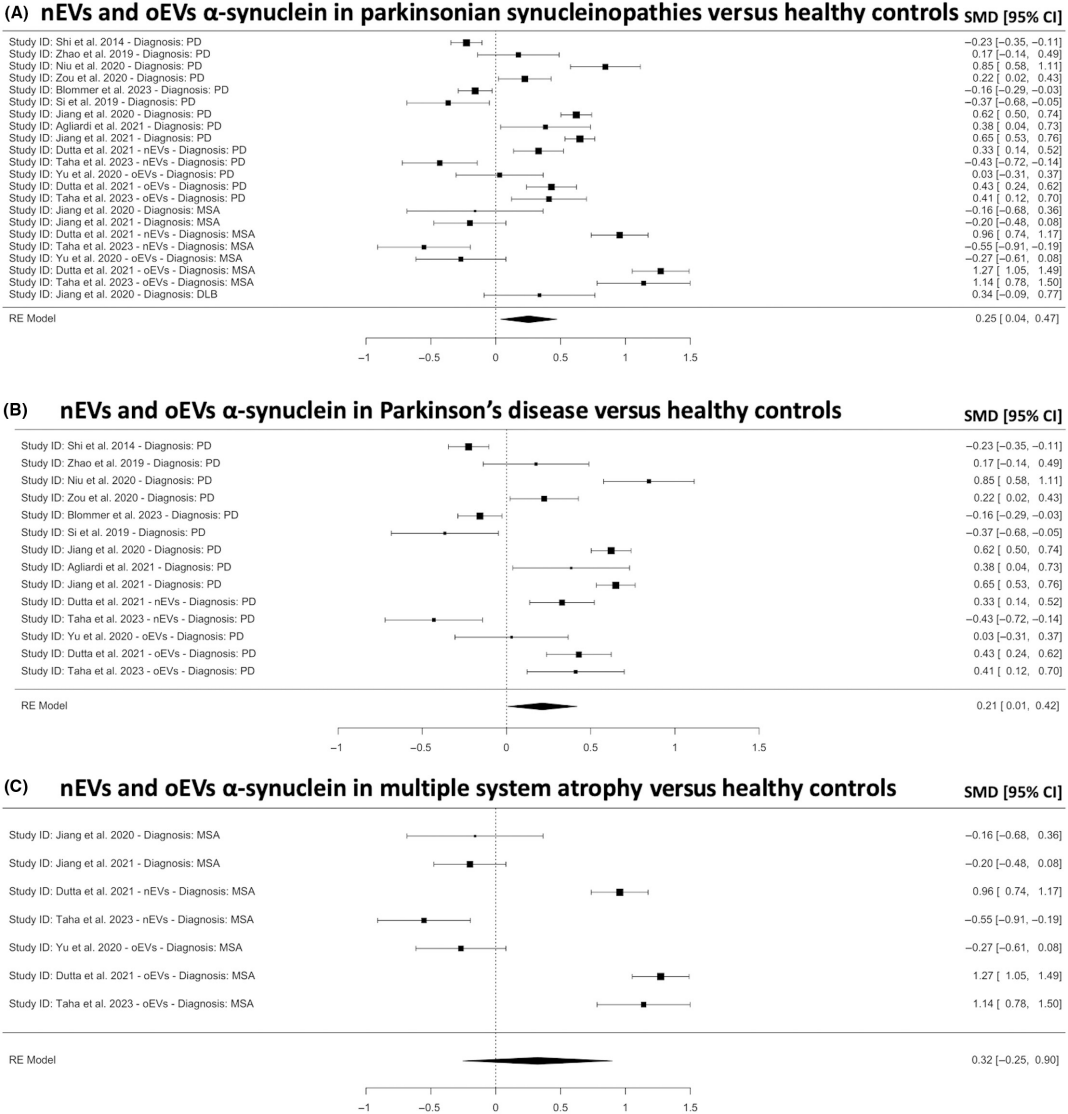
Meta‐analysis for the combination of neuronal and oligodendroglial EVs (nEVs and oEVs) α‐synuclein in (A) PD, MSA and DLB versus HCs, (B) PD versus HCs and (C) MSA versus HCs. A positive or negative SMD indicates higher or lower α‐synuclein, respectively. CI, confidence intervals; DLB, dementia with Lewy body; HC, healthy controls; MSA, multiple system atrophy; PD, Parkinson's disease; SMD, standardized mean difference.

Interestingly, when combining nEVs and oEVs, α‐syn showed statistically higher concentrations in synucleinopathies versus HCs (*k* = 22, SMD = 0.25; 95% CI 0.04, 0.47 *p* = 0.021; Figure 3A). This effect remained significant only for PD (*k* = 14, SMD = 0.21; 95% CI 0.01, 0.42 *p* = 0.021; Figure 3B) but not for MSA (*k* = 7, SMD = 0.32; 95% CI −0.25, 0.90; *p* = 0.27; Figure 3C). Separating the analyses for patients with PD versus HCs by medium (Figure S3A,B) or antibody (Figure S4A,B) abrogated this relationship (*p* > 0.05).

**FIGURE 4:**
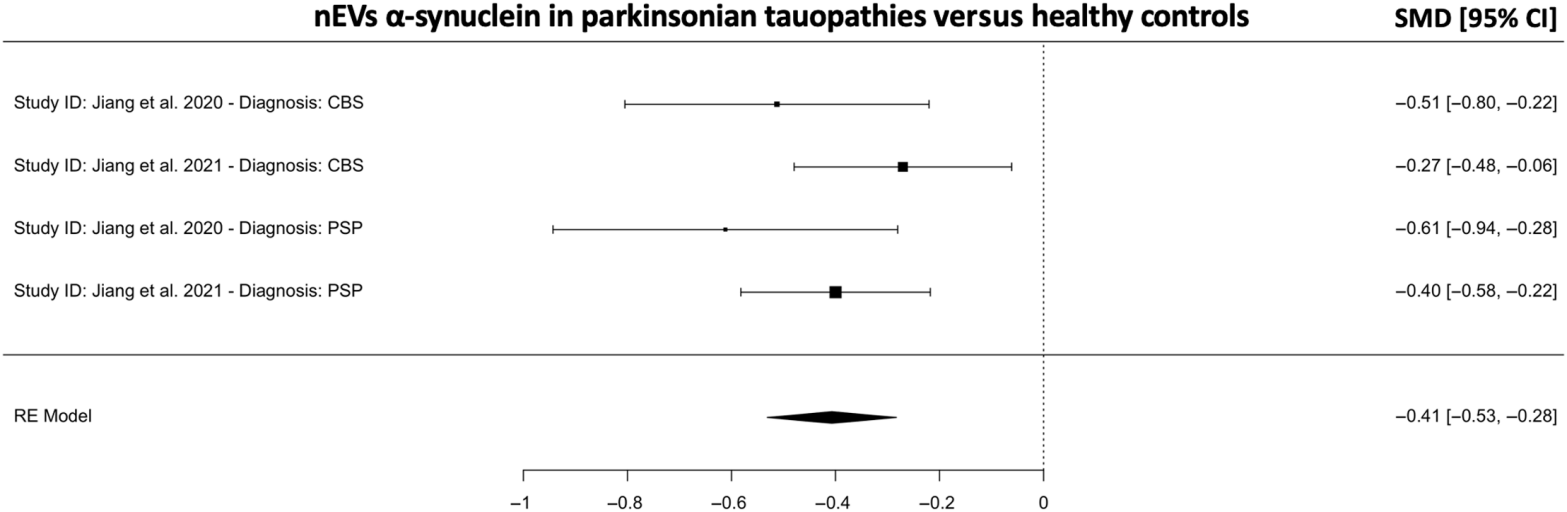
Meta‐analysis for neuronal EVs (nEVs) α‐synuclein in CBS and PSP versus HCs. A negative SMD indicates lower nEVs α‐synuclein concentrations in CBS and PSP versus HCs. CBS, corticobasal syndrome; CI, confidence intervals; HC, healthy controls; PSP, progressive supranuclear palsy; SMD, standardized mean difference.

Lastly, and surprisingly, a meta‐analysis revealed that nEVs α‐syn was found to be lower in the Parkinsonian tauopathies (PSP and CBS) versus HCs (*k* = 4, SMD = −0.41; 95% CI −0.53, 0.28; *p* < 0.001; Figure 4) with low heterogeneity (9.7%).

Though inspection of Funnel plots in all analyses suggested the possibility of publication bias, Begg's rank correlational and Egger's regression tests revealed no such significance (*p* > 0.05). In all analyses, except for PSP and CBS versus HCs, heterogeneity was deemed to be high (≥88.0%), and sensitivity analyses were conducted to exclude studies with high influence. However, removal of any such studies did not influence the significance, direction of the results or decrease the heterogeneity by a large margin, likely indicating that the results are robust.

Meta‐regression analyses revealed that age, gender, disease duration, motor impairment severity and cognition were not predictors of nEVs and/or oEVs α‐syn concentrations (*p* > 0.05). The above results suggest that combined nEVs and oEVs α‐syn may be a good marker for patients with PD versus HCs. It is also possible that nEVs α‐syn is a good marker for PSP and CBS versus HCs, though the interpretation is limited as the meta‐analyses included only two studies from the same research group.^18^, ^19^

### 3.3 nEVs α‐syn: Synucleinopathy versus synucleinopathy

PD and DLB have overlapping features, making diagnosis challenging. The key factor in distinguishing between them is the onset of dementia symptoms in relation to motor impairments. If dementia appears within a year of motor problems, it aids in differentiating DLB from PD. However, MSA presents as a more severe condition with a distinct pathological background, setting it apart from PD and DLB.

**FIGURE 5:**
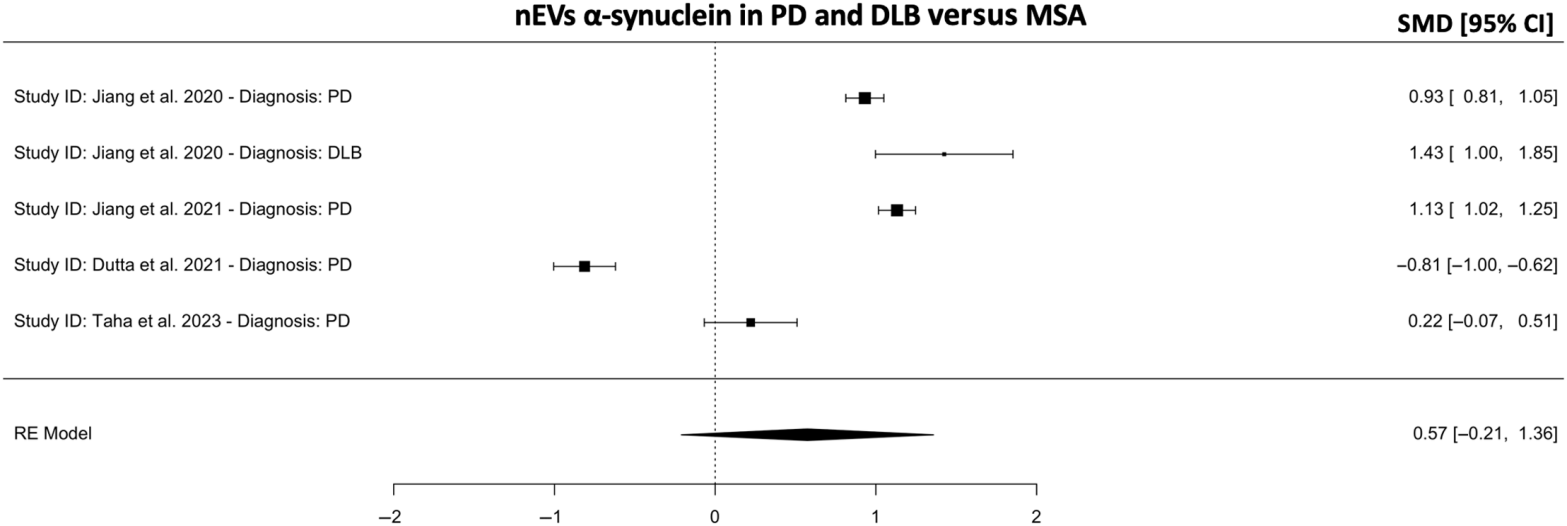
Meta‐analysis for neuronal EVs (nEVs) α‐synuclein in PD and DLB vs. MSA. A positive or negative SMD indicates higher or lower nEVs α‐synuclein concentrations in PD or DLB versus MSA, respectively. CI, confidence intervals; DLB, dementia with Lewy body; MSA, multiple system atrophy; PD, Parkinson's disease; SMD, standardized mean difference.

Therefore, we further asked whether nEVs α‐syn is different in PD and DLB versus MSA. The meta‐analysis revealed no significant differences, though the SMD trended toward a positive value (*k* = 6, SMD = 0.57; 95% CI −0.21, 1.36; *p* = 0.15; Figure 5) with high heterogeneity (98.8%). The results remained the same for PD versus MSA alone. As the number of studies included is small (*n* = 4), we did not conduct sensitivity analyses or meta‐regressions. As only one study measured nEVs α‐syn in DLB,^19^ no separate analysis for DLB versus MSA was conducted. The results remained unchanged using subgroup analyses.

### 3.4 nEVs α‐syn: Parkinsonian synucleinopathy versus tauopathy

**FIGURE 6:**
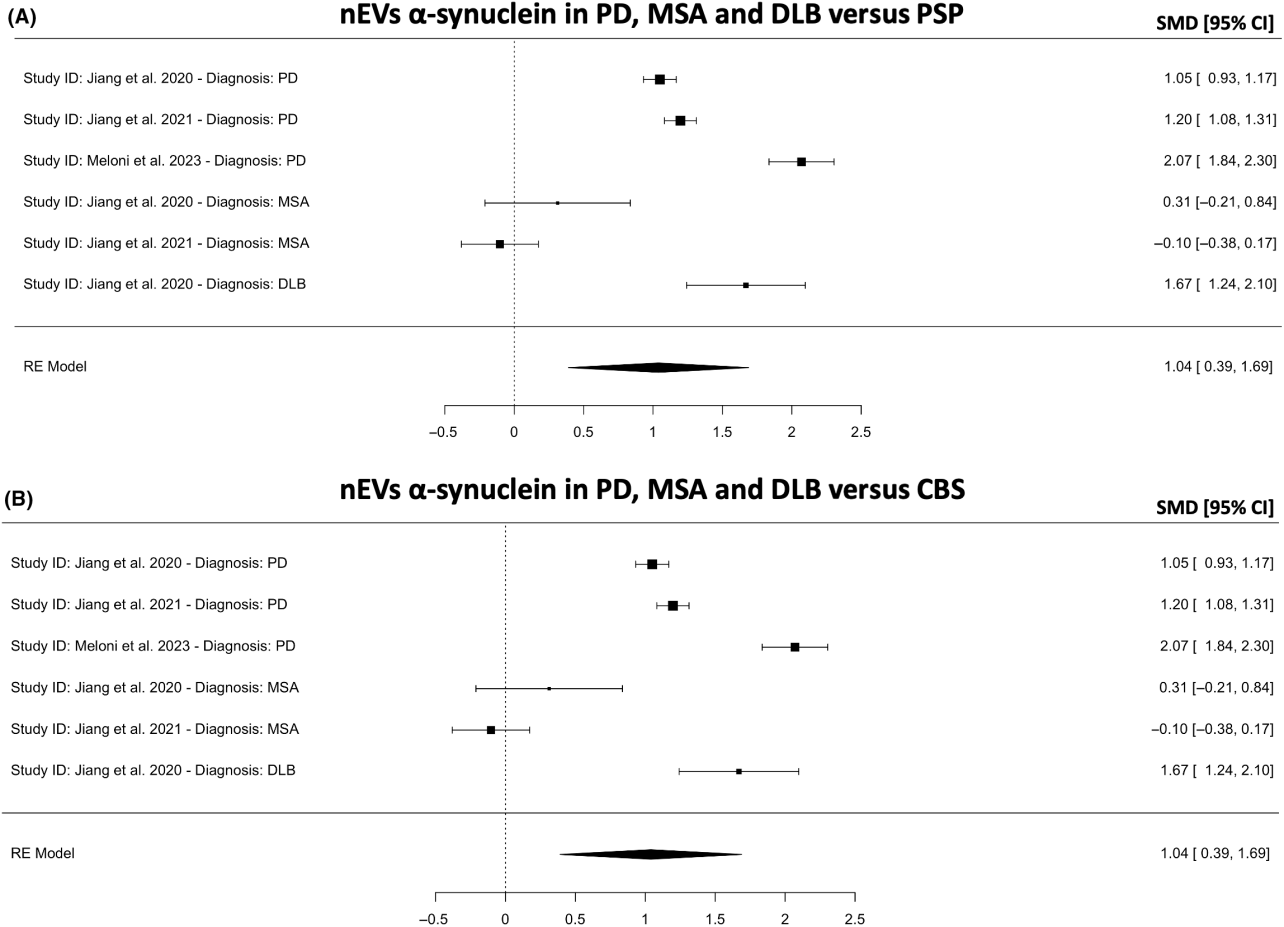
Meta‐analysis for neuronal EVs (nEVs) α‐synuclein in (A) PD, MSA and DLB vs PSP and (B) PD, MSA and DLB versus CBS. A positive or negative SMD indicates higher or lower concentrations of α‐synuclein, respectively. CBS, corticobasal syndrome; CI, confidence intervals; DLB, dementia with Lewy body; MSA, multiple system atrophy; PD, Parkinson's disease; PSP, progressive supranuclear palsy; SMD, standardized mean difference.

As α‐syn pathology mainly affects one of three synucleinopathies: PD, DLB or MSA, we further investigated whether nEVs α‐syn found in patients with a synucleinopathy differed from those who have an atypical Parkinsonian tauopathy (PSP or CBS). All studies included in this analysis used a similar antibody clone (anti‐L1CAM clone UJ127). The meta‐analysis revealed that nEVs α‐syn is higher only in PD and DLB versus PSP (*k* = 6, SMD = 1.04; 95% CI 0.39, 1.69; *p* = 0.0017; Figure 6A) and CBS (*k* = 6, SMD = 0.87; 95% CI 0.27, 1.47; *p* = 0.0020; Figure 6B). This is to be expected as the above meta‐analyses suggest that nEVs and oEVs α‐syn is significantly higher in PD versus HCs but nEVs α‐syn is significantly lower in PSP and CBS versus HCs. The heterogeneity was high, but sensitivity analyses revealed that no study was influential, indicating that the results are robust.

### 3.5 oEVs α‐syn: Parkinson's disease, multiple system atrophy and healthy controls

**FIGURE 7:**
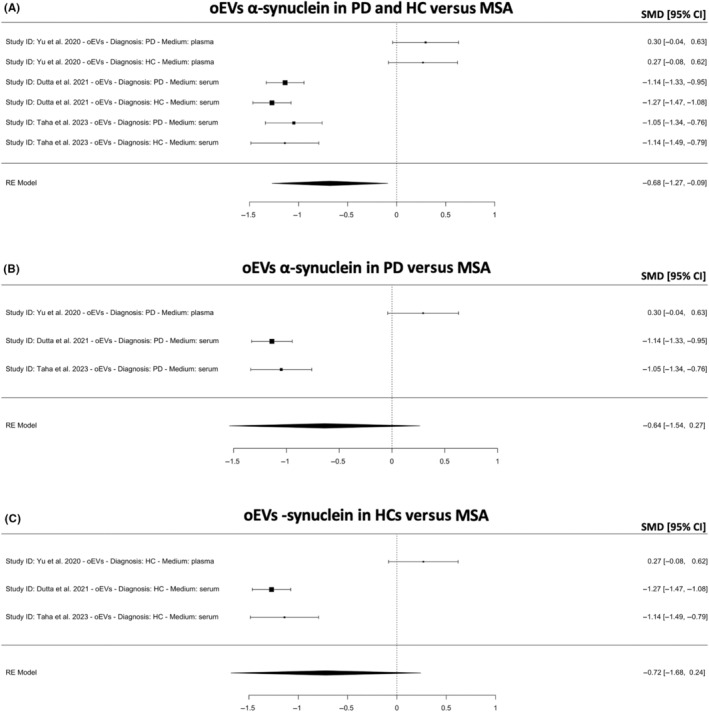
Meta‐analysis for oligodendroglial EVs (oEVs) α‐synuclein in (A) PD and HC versus MSA, (B) PD versus MSA and (C) HC versus MSA. A positive or negative SMD indicates higher or lower concentrations of α‐synuclein, respectively. CI, confidence intervals; DLB, dementia with Lewy body; HC, healthy controls; MSA, multiple system atrophy; PD, Parkinson's disease; SMD, standardized mean difference.

As oligodendroglial pathology and α‐syn aggregates (i.e., GCIs) are exclusive to MSA, three studies have quantified the concentration of α‐syn in oEVs in patients with PD, MSA or HCs in hopes of differentiating PD or HCs from MSA.^17^, ^24^, ^25^ The meta‐analysis revealed that oEVs α‐syn is lower in patients with PD and HCs combined versus patients with MSA (*k* = 6, SMD = −0.68; 95% CI −0.68, 0.09; *p* = 0.023; Figure 7A). Separate meta‐analyses comparing oEVs α‐syn in patients with PD versus MSA revealed that oEVs α‐syn did not differ between the two diseases (*k* = 3, SMD = −0.64; 95% CI −1.54, 0.27; *p* = 0.17; Figure 7B) or between HCs versus patients with MSA (*k* = 3, SMD = −0.72; 95% CI −1.68, 0.24; *p* = 0.14; Figure 7C). The likely explanation for this contradiction is that two studies from one research group^17^, ^24^ reported higher oEVs α‐syn concentrations in patients with MSA while one study from another research group^25^ reported lower oEVs α‐syn concentrations in patients with MSA. These results highlight oEVs α‐syn as controversial. Combining nEVs α‐syn with oEVs α‐syn revealed similar results.

Further, because MSA is more a severe disease than PD, and is usually distinguishable from HCs, while the accurate diagnosis of PD in early stages from HCs is more challenging, we asked whether oEVs α‐syn in patients with MSA or HCs is different than patients with PD. The meta‐analysis revealed no such differences when MSA and HC were combined (*k* = 3, SMD = 0.17; 95% CI −0.42, 0.76; *p* = 0.57; Figure S5).

**FIGURE 8:**
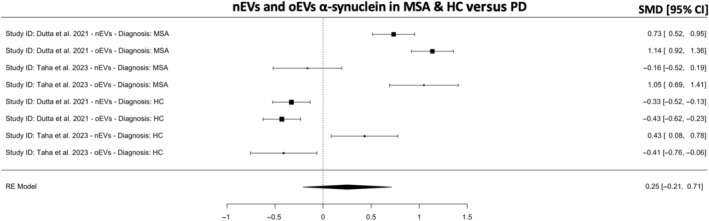
Meta‐analysis for neuronal and oligodendroglial EVs (oEVs) α‐synuclein in MSA and HC versus PD. A positive and negative SMD indicates higher or lower concentrations of α‐synuclein, respectively. CI, confidence intervals; DLB, dementia with Lewy body; HC, healthy controls; MSA, multiple system atrophy; PD, Parkinson's disease; SMD, standardized mean difference.

Two studies have suggested that a combination of nEVs and oEVs α‐syn is more useful than either measurement alone in separating patients with PD and HCs from MSA.^17^, ^24^ A meta‐analysis combining nEVs and oEVs α‐syn suggest that this is not the case (*k* = 8, SMD = 0.25; 95% CI −0.21, 0.71; *p* = 0.28; Figure 8 **)** even when combined with Yu et al.^25^ We did not perform sensitivity analyses or meta‐regressions due to the small number of studies (*n* = 3).

## 4 DISCUSSION

The accurate early diagnosis of Parkinsonian synucleinopathies: PD, MSA, and DLB and tauopathies: PSP and CBS, is crucial for several reasons. First, these disorders present with overlapping clinical symptoms despite clear differences in the pathophysiology of diseases, making accurate diagnosis challenging. This hinders the timely and accurate initiation of targeted therapeutic interventions, which may slow disease progression and improve patients' quality of life. Second, the ability to distinguish between these disorders allows for better patient stratification in clinical trials, leading to the development of more effective and tailored treatment strategies. Third, early diagnosis provides patients and their families with the information necessary to make informed decisions about end‐of‐life care and to plan accordingly. Therefore the use of non‐invasive or minimally invasive diagnostic approaches, such as biomarkers found in blood CNS‐originating EVs, has been popular as it contains the potential to reduce patient discomfort and minimize the risks associated with more invasive procedures by providing a window into the brain's biochemistry.^12^, ^15^, ^17^, ^18^, ^19^, ^21^, ^23^, ^24^

In this systematic review and meta‐analysis, we aimed to gather conclusive evidence regarding blood‐isolated nEVs and oEVs‐associated α‐syn proteoform levels in patients with a Parkinsonian disorder (PD, MSA, DLB, PSP or CBS) and HCs. In the 13 studies included, the results suggest that combined nEVs and oEVs α‐syn in patients with PD is higher than HCs (SMD = 0.21; *p* = 0.021; Figure 3B), though oligodendrocytes are not known to be affected in PD and a plausible mechanism is not known. In contrast, patients with PSP and CBS had lower nEVs α‐syn versus HCs (SMD = −0.41; *p* < 0.001; Figure 4). It is important to note, however, that the analyses with PSP and CBS versus HCs included only two studies from the same research group,^18^, ^19^ and as such, the reproducibility and generalizability are limited. Further, nEVs α‐syn was found to be higher in patients with PD versus PSP (SMD = 1.04; *p* = 0.0017; Figure 6A) and CBS (SMD = 0.87; *p* = 0.0020; Figure 6B), which is supported by the findings of individual studies.^18^, ^19^, ^21^ However, the analysis included only three studies and two of those studies were from the same research group.^18^, ^19^

Though oligodendrocytes α‐syn pathophysiology is believed to be exclusive to MSA, the results indicate that oEVs α‐syn is not different in patients with MSA versus patients with PD or HCs (Figure 7B,C). This is to be expected as only two studies from one research group^17^, ^24^ reported higher oEVs α‐syn concentrations in patients with MSA while one study from another research group^25^ reported lower oEVs α‐syn concentrations in patients with MSA. The results suggest that the measurement of α‐syn in oEVs is not reliable and is contradictory to known pathophysiological mechanisms. Lastly, these two studies from the same research group^17^, ^24^ have suggested that combining α‐syn measurement in nEVs and oEVs distinguishes PD and HCs from MSA with high accuracy. However, this is contradicted by the present meta‐analyses, which show that this is not the case (Figure 8).

The above results clearly illustrate the need for more standardized and rigorous methodologies across CNS‐originating EVs biomarkers for the differential diagnosis of Parkinsonian disorders from one another or from HCs. Currently, the consistent failure of independent validation/replication plagues the field of biomarkers in CNS‐originating EVs for Parkinsonian disorders.^12^, ^15^, ^16^, ^17^, ^18^, ^19^, ^21^, ^22^, ^23^, ^24^, ^25^, ^26^, ^41^, ^58^, ^59^ Instead of spending time, effort and money on these measurements, investigators should be aiming at standardizing critical preanalytical factors before the isolation of CNS‐originating EVs including the choice of anticoagulation agent mixed with plasma, the time of preparation, centrifugation methodology, the nature of transport, number of freeze/thaw cycles, storage conditions, temperature, and the type of collection tube^50^, ^51^, ^52^, ^53^, ^54^, ^55^, ^56^ as well as after enrichment of CNS‐originating EVs. A systematic review for L1CAM + EVs (i.e., putative nEVs) by Gomes & Witwer^30^ highlighted major limitations in these studies in terms of rigorous reporting of preanalytical factors before isolation of EVs as well as issues with reproducibility across the studies. The present meta‐analysis further supports these findings for the majority of analyses.

Two recent meta‐analyses evaluated the levels of putative “exosomal” α‐syn in patients with PD.^27^, ^28^ Nila et al.^27^ suggest that α‐syn is higher in patients with PD compared to HCs; however, as seen in their Figure 2., the effect is driven by a single study,^60^ which is the only study that evaluated α‐syn in EVs isolated from saliva. Furthermore, this study grouped together α‐syn concentrations found in bulk EV preparations with those originating from the CNS, which is problematic because they are not comparable.^29^ The purity, content, size, and number of EVs isolated from similar mediums (i.e., plasma vs. serum) are known to be dependent on various preanalytical factors. Lastly, their analyses for α‐syn in CNS‐originating EVs seen in Figure 4A include six studies,^15^, ^17^, ^23^, ^26^, ^41^, ^61^ one of which did not appear to enrich for nEVs.^61^ On the other hand, Valencia et al.^28^ conducted a much smaller study and omitted crucial analyses for α‐syn in CNS‐originating EVs.

The present meta‐analysis offers several advantages over previous studies conducted by Nila et al.^27^ and Valencia et al.^28^ Firstly, it includes at least six studies not evaluated or included in the earlier analyses, providing unique insights into α‐syn in CNS‐originating EVs. Secondly, it examines EVs from different cell types (nEVs and oEVs) across a range of Parkinsonian disorders (PD, MSA, DLB, PSP, and CBS) and HCs, offering a more comprehensive understanding. Lastly, the current meta‐analysis takes into account key aspects known to influence α‐syn concentrations, such as the medium used for isolation (i.e., plasma vs. serum) and the antibody used for immunocapture of putative CNS‐originating EVs, further enhancing the robustness of the findings.

## 5 CONCLUSION

The need to establish reliable and minimally invasive diagnostic biomarkers for Parkinsonian disorders is crucial for targeted interventions, improved clinical trial patient stratification and informed end‐of‐life care planning. Biomarkers found in blood‐isolated CNS‐originating EVs may provide insight into the brain's biochemistry, and thus, have become a popular source for biomarker discovery. The present meta‐analysis, comprising 13 studies, suggests that nEVs α‐syn may have value for differentiating patients with PD from HCs only when combined with oEVs α‐syn, which contradicts the existing literature.^17^, ^24^, ^25^ Furthermore, the levels of nEVs α‐syn demonstrate higher values in patients with PD compared to those with PSP and CBS. It is important to note, however, that these findings are based on three studies, two of which were conducted by the same research group.^18^, ^19^ On the other hand, nEVs and oEVs α‐syn have been suggested to differentiate patients with PD from MSA with high accuracy, yet the present meta‐analysis contradicted this particular finding.

Ultimately, this meta‐analysis highlights the necessity for standardized blood collection and EV isolation techniques/methodologies and underscores the importance of exploring additional biomarkers using the same approach such as Clusterin^19^ or a specific microRNA such as Linc‐POU3F3,^41^ as well as considering other biofluids such as cerebrospinal fluid,^62^ urine,^63^ skin^64^ stool^65^ or tears.^66^ It also suggests the exploration of other techniques for biomarker discovery such as protein seeding assays,^20^, ^67^ measurements of neurophysiological factors^68^ or kinematic fluctuations,^69^ among other potential approaches.

## AUTHOR CONTRIBUTIONS

Literature search, conception, design and writing of manuscript and approval of final draft—Hash Brown Taha. Data collection—Hash Brown Taha and Shomik S. Ati.

## CONFLICT OF INTEREST STATEMENT

The authors declare no conflict of interest.

## Supporting information


Data S1:

## Data Availability

All data, analyses, and their respective code are available from the corresponding author upon request.
