# Supplementary material for: Evaluation of α‐synuclein in CNS‐originating extracellular vesicles for Parkinsonian disorders: A systematic review and meta‐analysis
Source: CNS Neurosci Ther. 2023 Jul 7;29(12):3741–55. doi: 10.1111/cns.14341 (PMC10651986; doi:10.1111/cns.14341)
Supplement: Supplementary file 1 — Data S1: [file CNS-29-3741-s001.docx]

**Supplementary information**

**Table S1.** Search strategy.

| **PubMed** | ((((((((((((((((((((Parkinson's disease) OR (Lewy body disease)) OR (Lewy body dementia)) OR (Dementia with lewy body)) OR (Parkinson's disease dementia)) OR (Multiple system atrophy)) OR (Supranuclear Palsy)) OR (Corticobasal syndrome)) OR (corticobasal degeneration)) OR (Parkinsonian Disorders)) OR (alpha-synuclein)) AND (Exosome OR Extracellular vesicle OR EV)) AND (Blood OR Serum OR Plasma OR Cerebrospinal fluid)) AND Neuron) AND synuclein) |
| --- | --- |
| **Embase** | (('parkinson disease':ti,ab,kw OR 'lewy body disease':ti,ab,kw OR 'lewy body dementia':ti,ab,kw OR 'dementia with lewy body':ti,ab,kw OR 'multiple system atrophy':ti,ab,kw OR 'supranuclear palsy':ti,ab,kw OR 'corticobasal syndrome':ti,ab,kw OR 'corticobasal degeneration':ti,ab,kw OR 'parkinsonian disorders':ti,ab,kw) AND 'exosome':ti,ab,kw OR 'extracellular vesicle':ti,ab,kw OR 'ev':ti,ab,kw) AND 'synuclein':ti,ab,kw |

**Table S2.** Rubric for QUADAS-2.

| DOMAIN | DOMAIN 1  Patient selection | DOMAIN 2  Index test(s) | DOMAIN 3  Reference standard | DOMAIN 4  Flow and timing |
| --- | --- | --- | --- | --- |
| Description | Describe methods of patient selection.  Describe included patients (prior testing, presentation, intended use of index test and setting). | Describe the index test and how it was conducted and interpreted. | Describe the reference standard and how it was conducted and interpreted. | Describe any patients who did not receive the index test(s) and/or reference standard or who were excluded from the 2x2 table (refer to flow diagram).  Describe the time interval and any interventions between index test(s) and reference standard. |
| Signaling questions (*yes/no/unclear*) | Was a case-control design avoided?  Was a consecutive or random sample of patients enrolled?  Did the study avoid inappropriate exclusions? | Were the index test results interpreted without knowledge of the results of the reference standard?  If a threshold was used, was it pre-specified? | Is the reference standard likely to correctly classify the target condition?  Were the reference standard results interpreted without knowledge of the results of the index test? | Was there an appropriate interval between index test(s) and reference standard?  Did all patients receive a reference standard?  Did patients receive the same reference standard?  Were all patients included in the analysis? |
| Overall judgement | Included studies only were considered eligible if they determined the levels of α-syn in CNS-originating extracellular vesicles (either neuronal (nEVs) or oligodendroglial (oEVs) in at least Parkinson’s disease (low risk of bias) and one other parkinsonian disorder or healthy controls  Unclear risk of bias was considered in the absence of information on consecutive patient enrollment.  High risk of bias was based on the absence of information on consecutive patient enrollment and any unexplained or suspected exclusions. | Even though knowledge of the diagnosis may affect the interpretation of the diagnostic test results, measuring α-syn in nEVs or oEVs is an objective method that should not be influenced by the diagnosis.  This is considered a low risk of bias, even if blinding was not used. | To diagnose PD, the standard used was the United Kingdom Parkinson's Disease Society Brain Bank or the MDS clinical diagnostic criteria were used. To diagnose MSA, the second consensus statement on the diagnosis of multiple system atrophy was used To diagnose DLB, the fourth consensus report of the DLB consortium was used. To diagnose PSP, the NINDS-SPSP International workshop or the movement disorder society criteria were used To diagnose CBS, the criteria for the diagnosis of corticobasal degeneration were used. The clinical diagnoses were established before the index test (low risk of bias).  If the diagnosis was based on symptoms/signs without consultation of the diagnostic criteria listed above, the study was rated as “unclear risk of bias”. | All patients were classified according to the appropriate diagnostic criteria (see Domain 3).  Low risk of bias was considered if all the questions were answered “yes”.  Unclear risk of bias was considered if they did not cover the time interval between clinical diagnosis and index test.  High risk of bias was considered if the study excluded any of the participants from the analysis. |
| Concerns regarding applicability  (*High/low/unclear*) | Are there concerns that the included patients do not match the review question? | Are there concerns that the index test, its conduct, or interpretation differs from the review question? | Are there concerns that the target condition as defined by the reference standard does not match the review question? | Could the patient flow have introduced bias? |
| Applicability:  Overall judgement | As reported in the inclusion and eligibility criteria, the clinical diagnoses for the diseases were based on established diagnostic criteria (see Domain 3). Therefore, all studies were rated as “low concern/high applicability” | If the protein concentrations were determined using a standard calibration curve, the risk of bias was considered low. If this information was not provided, the risk of bias was deemed unclear.  The usage of in-house developed tests was considered “high concern/low applicability” | Because all the studies used internationally recognized criteria for their assessments, the risk of bias was considered low. | NA |

**Table S3.** Risk of Bias assessment according to the QUADAS-2 per study included in the meta-analyses.

|  | Bias | | | | Applicability concern | | | | |  |
| --- | --- | --- | --- | --- | --- | --- | --- | --- | --- | --- |
|  | Patient selection | Index text | Reference standard | | | Flow and timing | Patient selection | Index text | Reference standard | |
| Shi et al. 2014 | U | L | | L | | L | L | H | L | |
| Zhao et al. 2019 | U | L | | L | | L | L | L | L | |
| Si et al. 2019 | U | L | | L | | L | L | L | L | |
| Jiang et al. 2020 | U | L | | L | | L | L | L | L | |
| Niu et al. 2020 | U | L | | L | | L | L | L | L | |
| Zou et al. 2020 | U | L | | L | | L | L | L | L | |
| Yu et al. 2020 | U | L | | L | | L | L | H | L | |
| Agliardi et al. 2021 | U | L | | L | | L | L | L | L | |
| Jiang et al. 2021 | U | L | | L | | L | L | L | L | |
| Duta et al. 2021 | U | L | | L | | L | L | L | L | |
| Blommer et al. 2023 | U | L | | L | | L | L | L | L | |
| Meloni et al. 2023 | U | L | | L | | L | L | L | L | |
| Taha et al. 2023 | U | L | | L | | L | L | H | L | |


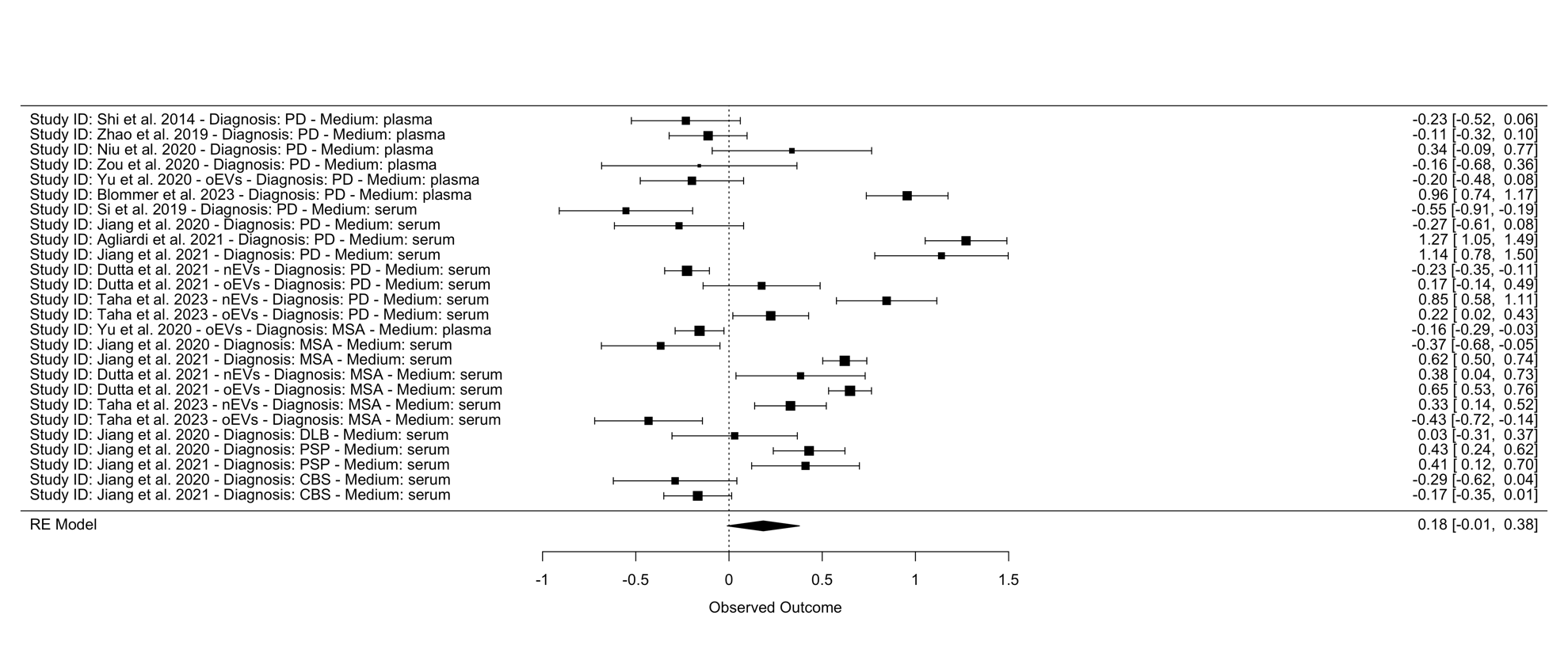


**Fig. S1.** **Meta-analysis for neuronal EVs (nEVs) and oligodendroglial EVs (oEVs) α-synuclein in Parkinsonian disorders vs. healthy controls.** A positive or negative SMD indicates higher or lower nEVs α-synuclein concentrations in a parkinsonian disorder vs. healthy controls, respectively. SMD – standardized mean difference; CI – confidence intervals; PD – Parkinson’s disease; MSA – multiple system atrophy; DLB – dementia with Lewy body; PSP – progressive supranuclear palsy; CBS – corticobasal syndrome; HC – healthy controls.


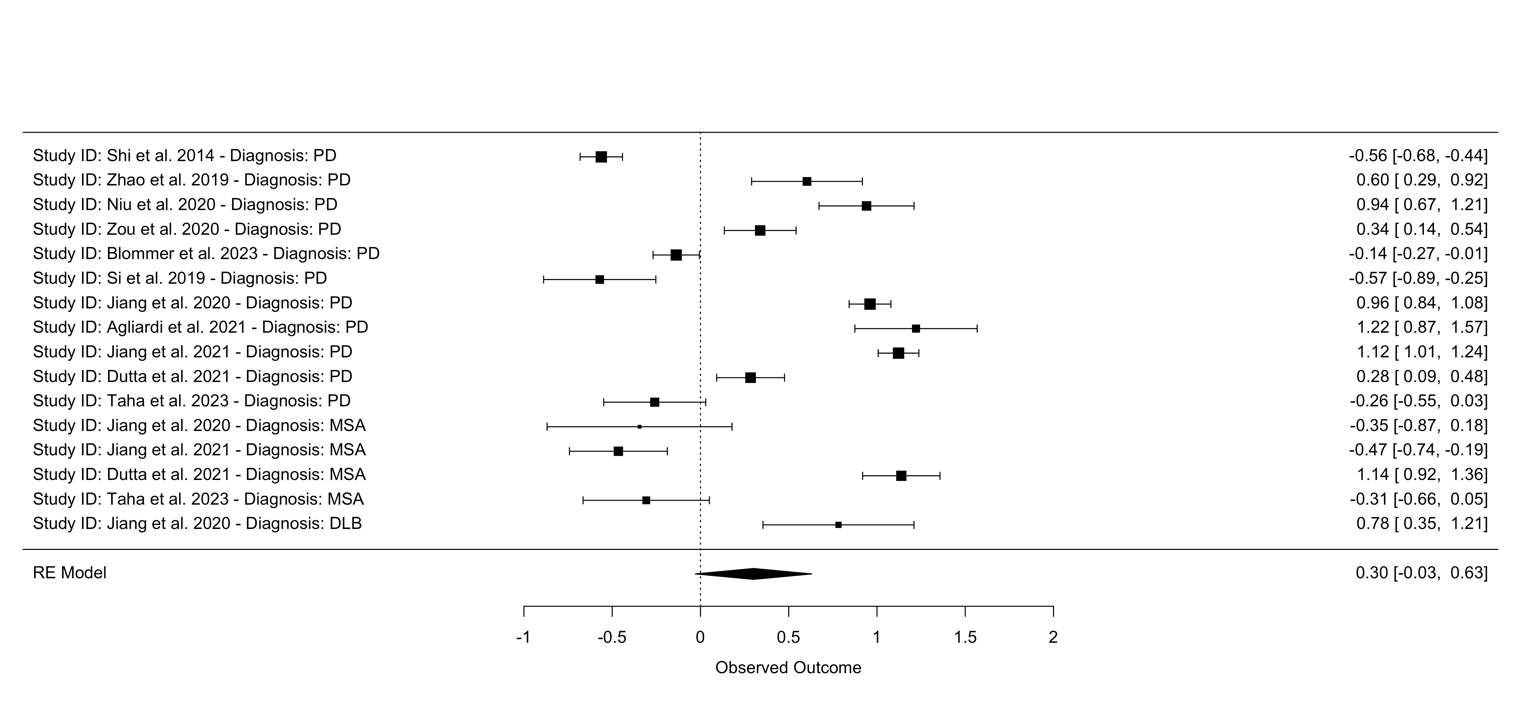


A

B


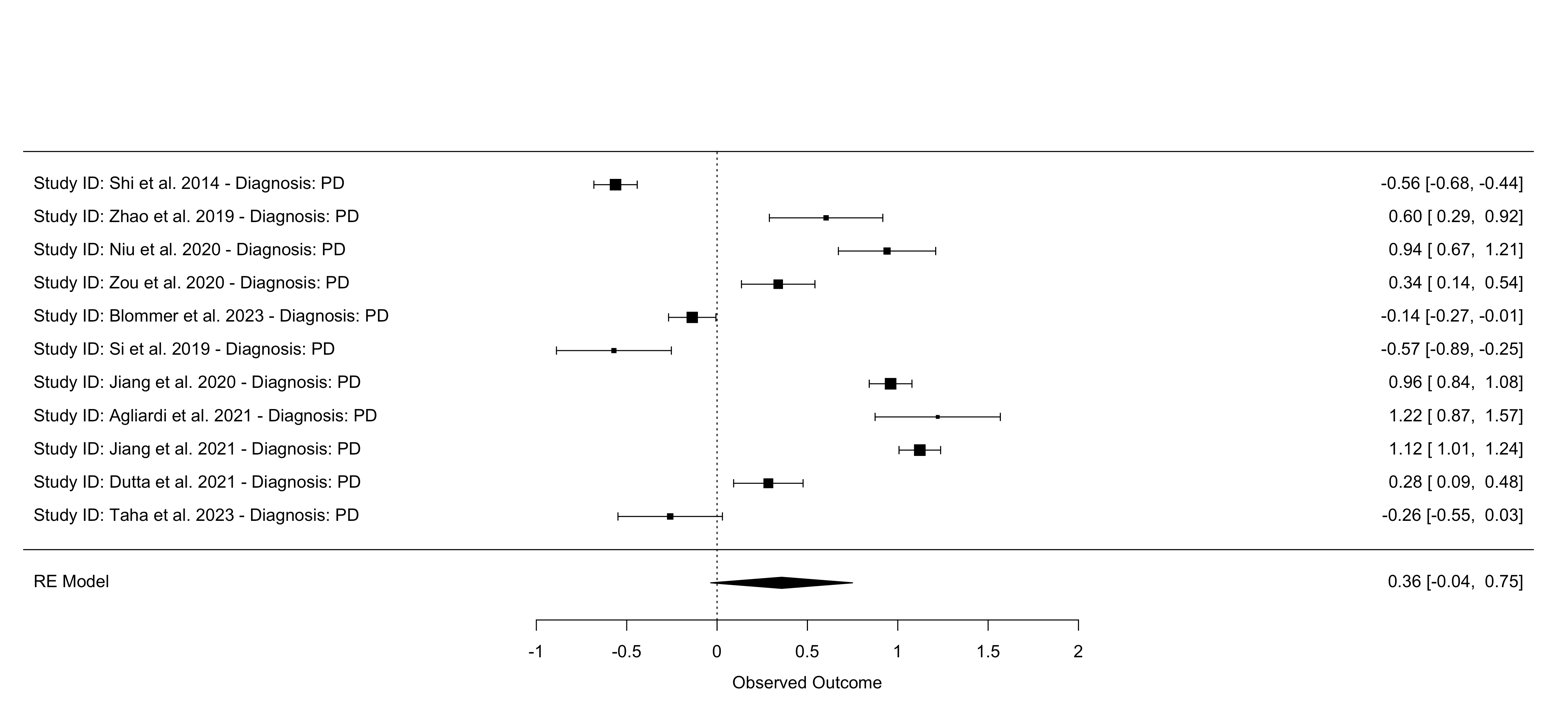


C


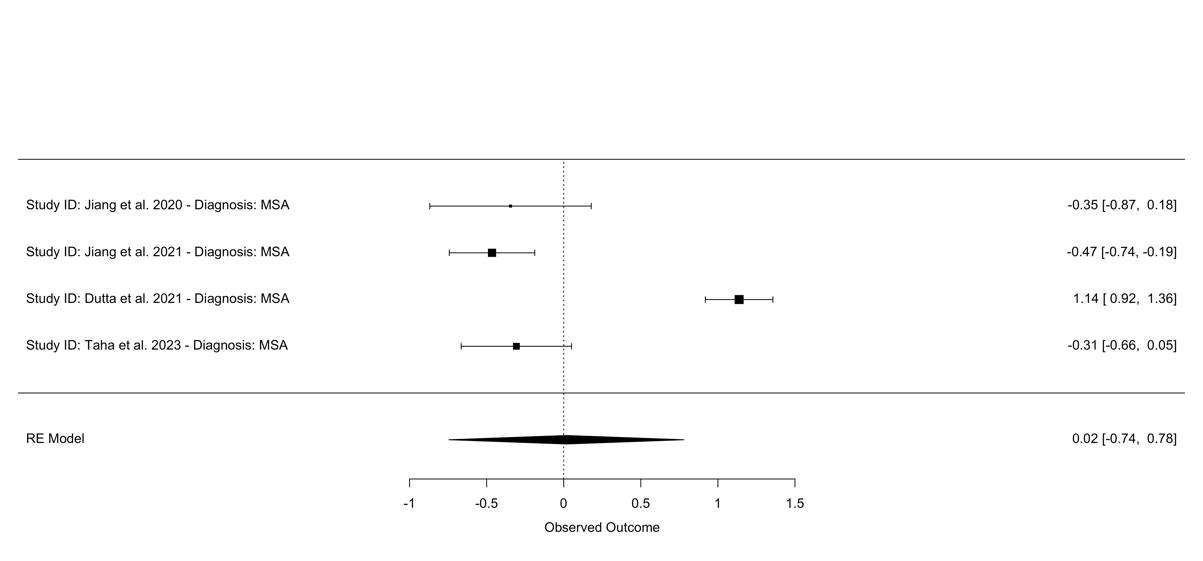


**Fig. S2.** **Meta-analysis for neuronal EVs (nEVs) α-synuclein in A.** PD, MSA, DLB vs. HCs, **B.** PD vs. HCs and **C.** MSA vs. HCs. A positive or negative SMD indicates higher or lower nEVs α-synuclein concentrations in a parkinsonian disorder vs. healthy controls, respectively. SMD – standardized mean difference; CI – confidence intervals; PD – Parkinson’s disease; MSA – multiple system atrophy; DLB – dementia with Lewy body; HC – healthy controls.


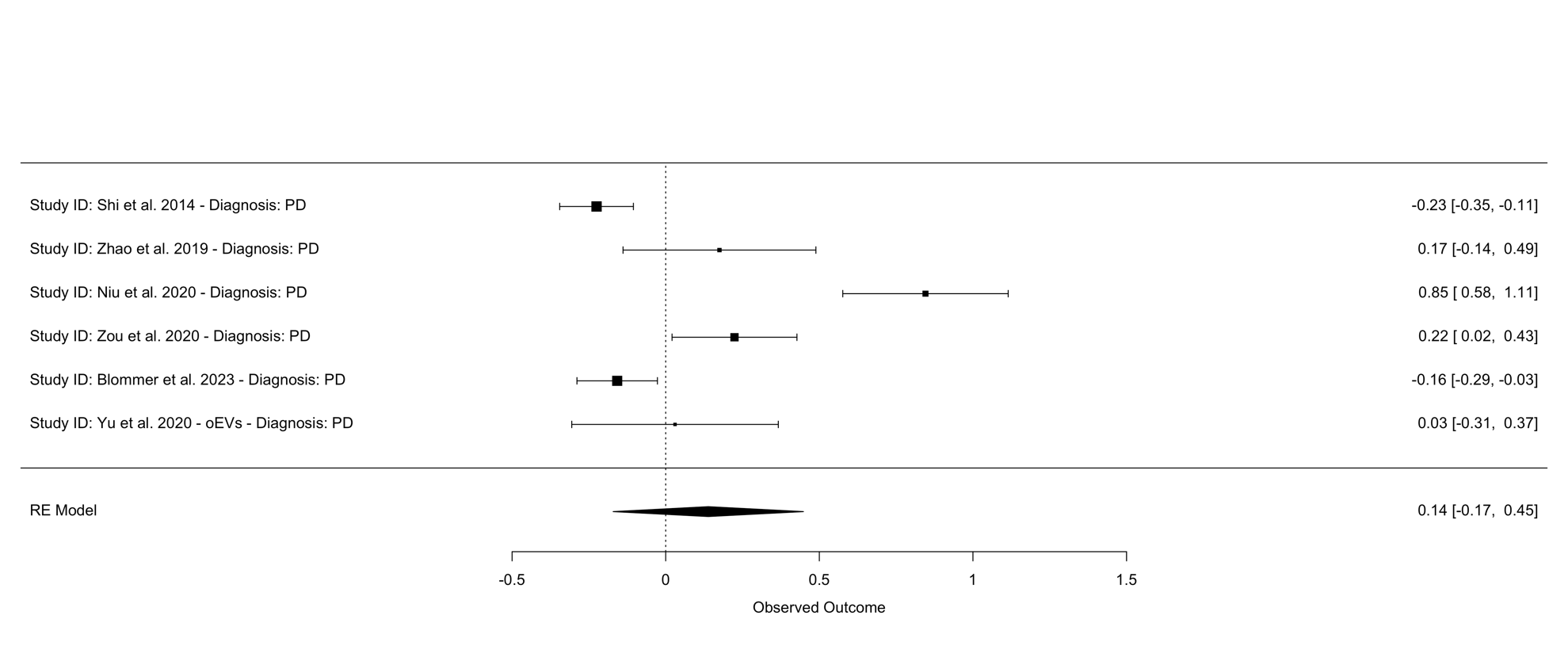

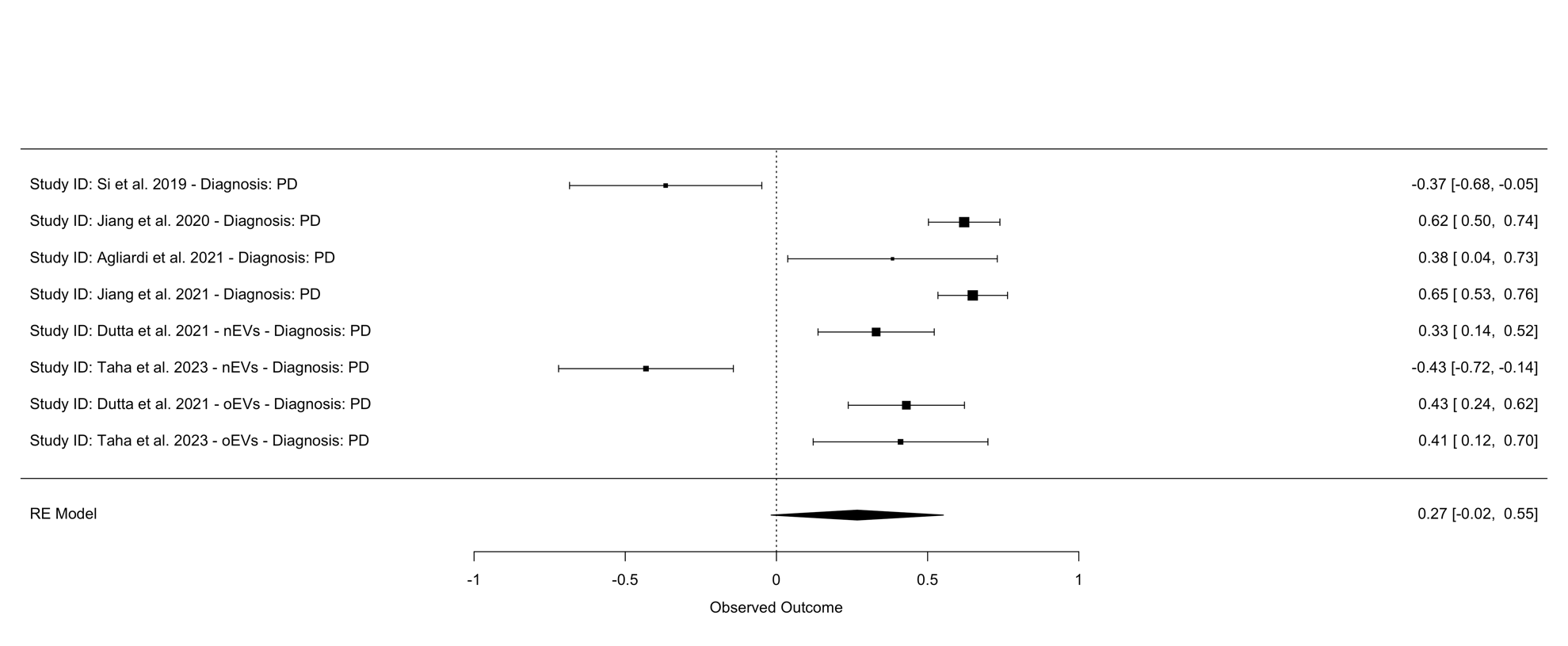
**Fig. S3.** **Meta-analysis for neuronal EVs (nEVs) α-synuclein in PD vs. HCs for A.** serum samples and **B.** plasma samples. A positive or negative SMD indicates higher or lower nEVs α-synuclein concentrations respectively. SMD – standardized mean difference; CI – confidence intervals; PD – Parkinson’s disease; HC – healthy controls.

B

A


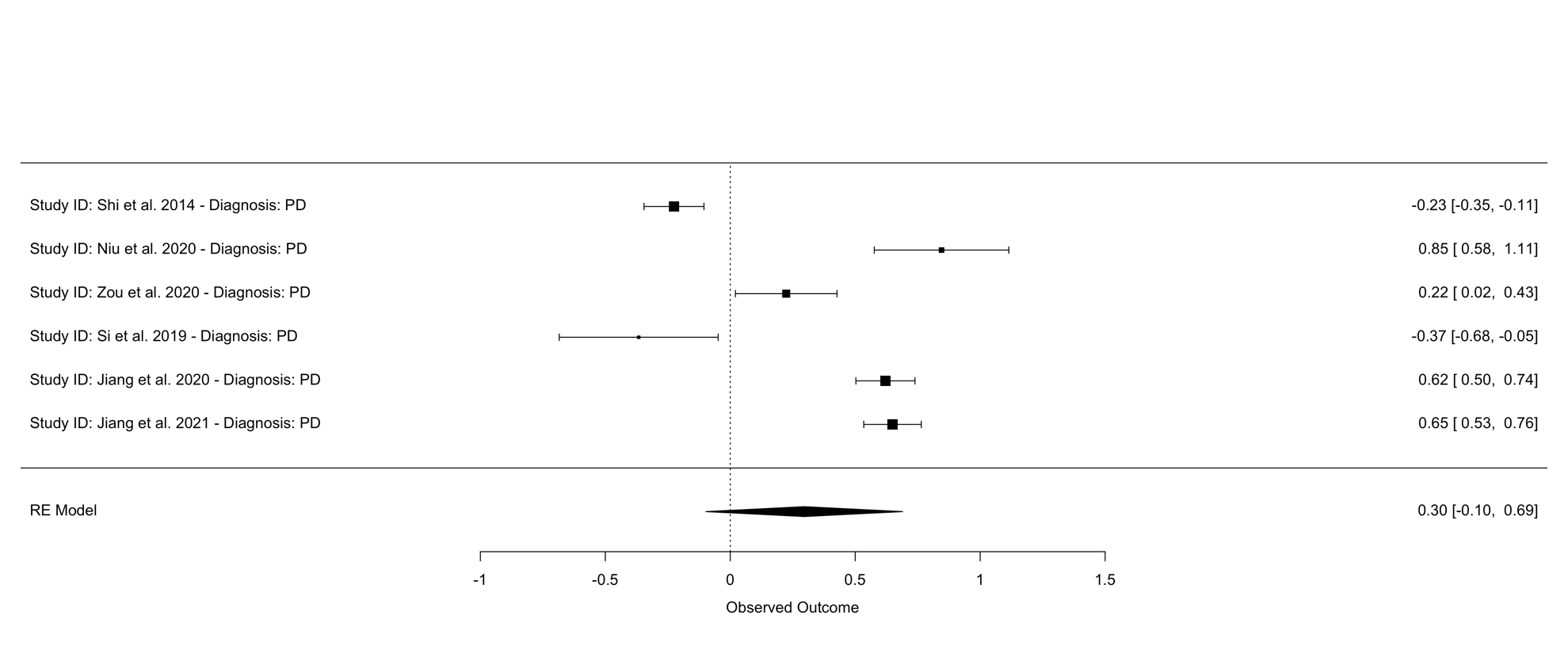


A

B

**Fig S4.** **Meta-analysis for neuronal EVs (nEVs) α-synuclein in PD vs. HCs for anti-L1CAM clone A.** 5G3 and **B.** UJ127. A positive or negative SMD indicates higher or lower nEVs α-synuclein concentrations, respectively. SMD – standardized mean difference; CI – confidence intervals; PD – Parkinson’s disease
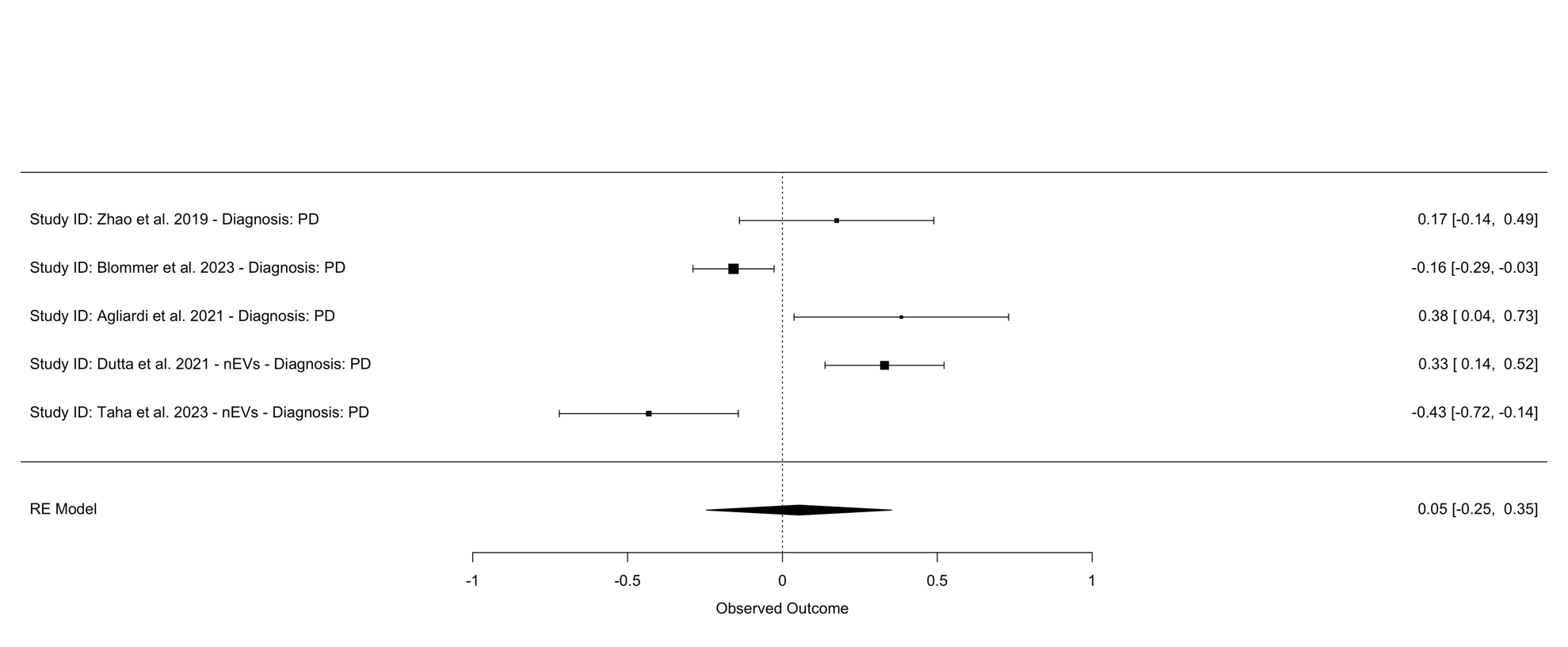
; HC – healthy controls.


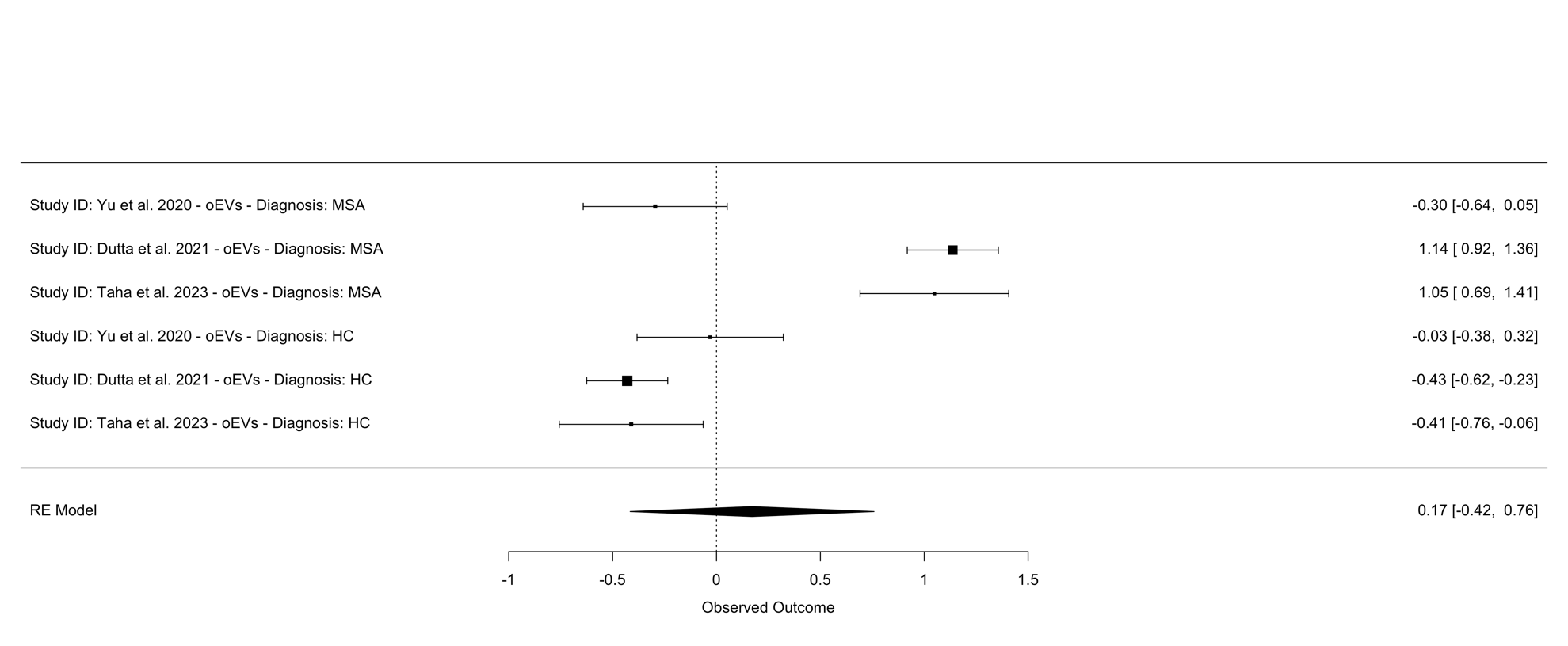


**Fig S5.** **Meta-analysis for oligodendroglial EVs (oEVs) α-synuclein in MSA and HC vs. PD.** A positive or negative SMD indicates higher or lower nEVs α-synuclein concentrations. SMD – standardized mean difference; CI – confidence intervals; MSA – multiple system atrophy; HC – healthy controls; PD – Parkinson’s disease.
